# Biogeography of Wood-Boring Crustaceans (Isopoda: Limnoriidae) Established in European Coastal Waters

**DOI:** 10.1371/journal.pone.0109593

**Published:** 2014-10-14

**Authors:** Luísa M. S. Borges, Lucas M. Merckelbach, Simon M. Cragg

**Affiliations:** 1 Institute of Marine Sciences, School of Biological Sciences, Portsmouth University, Eastney, Portsmouth, United Kingdom; 2 Helmholtz-Zentrum Geesthacht, Centre for Material and Coastal Research, Max-Planck-Straße 1, Geesthacht, Germany; Institute of Biochemistry and Biology, Germany

## Abstract

Marine wood-borers of the Limnoriidae cause great destruction to wooden structures exposed in the marine environment. In this study we collated occurrence data obtained from field surveys, spanning over a period of 10 years, and from an extensive literature review. We aimed to determine which wood-boring limnoriid species are established in European coastal waters; to map their past and recent distribution in Europe in order to infer species range extension or contraction; to determine species environmental requirements using climatic envelopes. Of the six species of wood-boring *Limnoria* previously reported occurring in Europe, only *Limnoria lignorum*, *L. quadripunctata* and *L. tripunctata* are established in European coastal waters. *L. carinata* and *L. tuberculata* have uncertain established status, whereas *L. borealis* is not established in European waters. The species with the widest distribution in Europe is *Limnoria lignorum*, which is also the most tolerant species to a range of salinities. *L. quadripunctata* and *L. tripunctata* appear to be stenohaline. However, the present study shows that both *L. quadripunctata* and *L. tripunctata* are more widespread in Europe than previous reports suggested. Both species have been found occurring in Europe since they were described, and their increased distribution is probably the results of a range expansion. On the other hand *L. lignorum* appears to be retreating poleward with ocean warming. In certain areas (e.g. southern England, and southern Portugal), limnoriids appear to be very abundant and their activity is rivalling that of teredinids. Therefore, it is important to monitor the distribution and destructive activity of these organisms in Europe.

## Introduction

The most economically important wood boring Crustacea in European waters belong to the Limnoriidae, isopods commonly known as gribbles. This family also includes species that bore into marine algae and seagrasses [1], [2]. Wood boring limnoriids evolved two key adaptations to use wood as substrate. The first was the ability to tunnel into wood for protection [3], and the second was to use wood as primary carbon source by producing endogenous enzymes that digest lignocellulose [4], [5]. The activity of limnoriids damages wooden man-made structures, such as piers, navigational dolphins, lock gates, and aquaculture facilities [6], [7]. The costs of the damage inflicted by limnoriids have never been evaluated globally, but we estimate it to be of the order of billions of Euros worldwide.

The economic impact and hazard posed by marine wood borers on the wooden dykes of The Netherlands was on the origin of the first scientific studies on these organisms in the 18^th^ century (e.g. [8]). However, these initial studies focused almost entirely on bivalves of the Teredinidae (shipworms). Although there is evidence that limnoriids were observed in these initial studies [9], they were probably not considered a threat to wooden structures. Indeed, only 60 years after the first studies on marine wood borers took place, the first wood boring limnoriid was described from Norway as *Cymothoa lignorum* (Rathke, 1799), now placed in the genus *Limnoria*. *Limnoria lignorum* was recognised as a wood-borer by Rathke [10], but a serious interest on its destructive activity started much later. In 1886, limnoriids were observed attacking wooden dykes of The Netherlands, an observation that prompted the intervention of a Committee of the Academy of Sciences of Amsterdam. It was found that *Limnoria lignorum*, the only limnoriid species known at the time, was widespread along the Dutch North Sea coast and in some places in the Zuider Zee (present day Lake IJssel). The hazard posed by these organisms to the integrity of the wooden dykes facings led to several studies encompassing the ecology, activity, and taxonomic position of *L. lignorum* (Hoek, 1893 in [9]).

In spite of these studies on *Limnoria lignorum* in The Netherlands, other economically important wood boring limnoriids were only described in middle twentieth century. The work of Holthuis [11] essentially launched the modern era of recognition of limnoriid diversity, when he described *L. quadripunctata* Holthuis, 1949, from the Dutch province of Zuid-Holland. The discovery of *L. tripunctata* Menzies, 1951, described from California, and also found in Europe (e.g. [12], [13], followed soon thereafter. It was also observed that the activity of *L. tripunctata* was leading to the premature failure of creosote-treated wooden structures, which are usually resistant to teredinids [14]. Interest in limnoriids increased again in the 1990s leading to the description of additional wood-boring species worldwide [15], [16].

The distribution of limnoriids is controlled by environmental and biological factors as well as the presence of wood [17]. The most important environmental factors controlling the distribution and survival of limnoriids are temperature and salinity [18], [19], [20]. Temperature influences the boring activity, the feeding rates of limnoriids [19], and it is particularly important during the reproductive and migratory season [18]. Salinity also plays an important role on feeding rates and distribution of limnoriids [19], [20], [21]. The limiting values of salinity seem to vary with temperature [20], but it was observed that limnoriids tend to occur in areas with salinity higher than 15 PSU [7], [22].

The life history strategy is also very important to explain the distribution of limnoriids. These organisms have low fecundity, iteroparity, and direct development [1]. Adult limnoriids tend to occur in pairs in tunnels, where copulation occurs. The fertilized eggs and juveniles develop in the brood pouch, a structure formed by the leaf-like extensions (oostegites) of the first four pair of peraeopods. The incubation time varies from 2 to 4 weeks depending on the species. After this period, the juveniles are released into the parental tunnels from where they tunnel out, excavating perpendicular tunnels [23], protected from adverse outside conditions by the parents [24]. Later, environmental conditions, mainly temperature, trigger the young-adults to leave the tunnels on a migratory journey to find and colonise fresh wood [18].

Information on the distribution of limnoriid species in European coastal waters is scarce and scattered in the literature and online databases. Therefore, the aims of the present study were: to collate data obtained from our field surveys in Europe over a ten-year period, with data from a comprehensive literature survey dating back to the 1900s; to use the data to map the recent and past distribution of limnoriid species in Europe, from which range expansion or contraction of species was detected; to determine their temperature and salinity requirements using climatic envelopes to help explaining changes in the range of species, as previously done for teredinids [25].

## Results

### Limnoriid species established in European coastal waters

**Figure 1:**
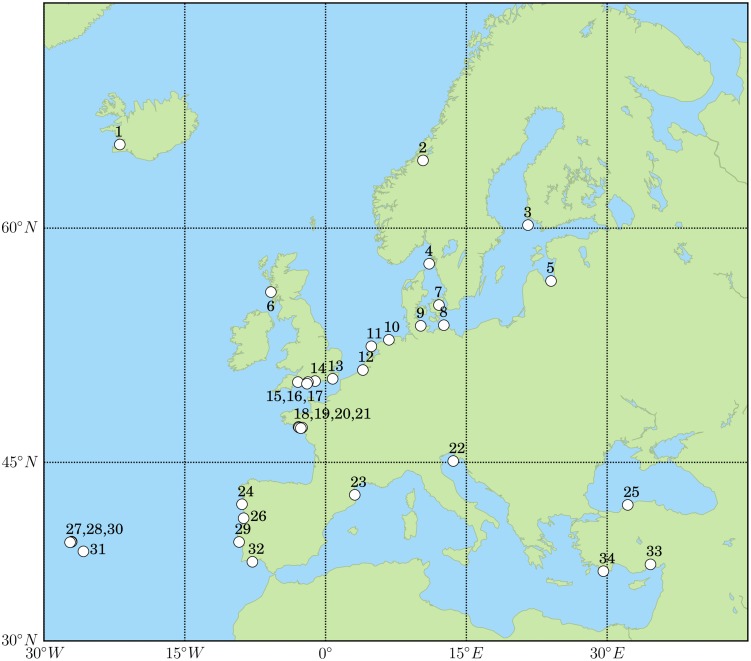
Sites surveyed between 2001 and 2011.

In our field surveys limnoriids were found in 19 out of the 34 sites surveyed (Fig. 1). They did not recruit to panels exposed in Riga, Latvia; Island of Jurmo, Finland; Kristineberg Marine Station, Sweden; estuary of the River Prerowstorm and Kiel, Germany; Haren and near Texel, The Netherlands; Rovinj, Croatia; Bartin and Ka, Turkey. Limnoriids were not found either in wooden structures surveyed in Rye, England or in wooden structures in Toulindac, Berder and Penerf, France.

**Figure 2:**
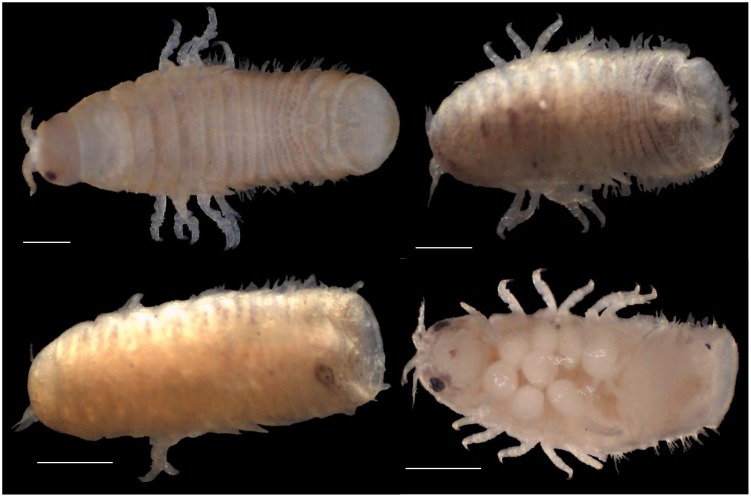
Specimens of *Limnoria lignorum*, *L. quadripunctata* and *L. tripunctata* collected in the field surveys. A) Dorsal view of preserved specimen of *L. lignorum*; B) dorsal view of preserved specimen of *L. quadripunctata*; C) dorsal view of preserved specimen of *L. tripunctata*; D) ventral view of preserved specimen of *L. tripunctata*. Scale bar = 0.5 mm.

**Figure 3:**
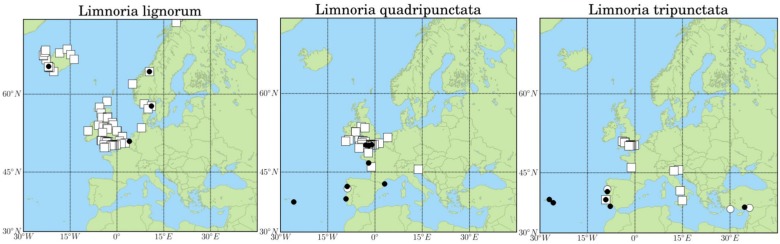
Past and present distribution of *Limnoria lignorum*, *L. quadripunctata* and *L. tripunctata* in European coastal waters. Squares represent data obtained from the literature before 2000; black circles represent data obtained from field surveys between 2001 and 2011; white circles represent data reported in the literature since 2000.

Three limnoriid species (Fig. 2) recruited to the panels in the long-term field survey (2002–2003), were found in local wooden structures during opportunistic surveys (Fig. 1, Table 1), and they were also reported in the literature (Table S1). *Limnoria lignorum* was found in the eastern north Atlantic and North Sea coasts, as far north as Reykjavik, Iceland, and as far south as Newton Ferrers, southern England. *Limnoria quadripunctata* and *Limnoria tripunctata* were found both in the Atlantic and Mediterranean coasts of Europe. The northern limit of *Limnoria quadripunctata*, was Ramsey, Isle of Man, whereas its southernmost limit was Mosteiros, São Miguel, Azores, Portugal. The northernmost occurrence of *Limnoria tripunctata* was Swansea, Wales and the southernmost limit was Finike, Turkey (Mediterranean Sea) (Fig. 3; Table S1). The species was also recently reported from North Africa, southern Mediterranean [26]. *Limnoria carinata* Menzies & Becker, 1957 and *Limnoria tuberculata* Sowinsky, 1884, were not found in our field surveys. *L. carinata* has so far only been found in La Spezia and the Gulf of Naples, Italy [27], while *L. tuberculata* was reported occurring in the Black Sea, Aegean Sea [28], [29], and southern England [35]. *Limnoria borealis* was reported occurring in Litladjúp, and off Thistilfjördur, Iceland, at depths of 223–260 m and 170–260 m, respectively. There was no information concerning the habitat where the specimens were found [30].

**Table 1:** Location of test sites.

Sites surveyed	Coordinates	Year(s) and duration	Type of structure
**1 Reykjavik- Iceland**	64.15; −21.90	2002/2003 (1 year)	wooden panels
**2 Trondheim- Norway**	63.41; 10.41	2002/2003 (1 year)	wooden panels
**3 Island of Jurmo- Finland**	60.15; 21.57	2002 (1 year)	wooden panels at shipwreck site
**4 Kristineberg Marine Biological Station- Sweden**	58.03; 11.05	2002/2003 (1 year)	wooden panels
**5 Riga- Latvia**	57.06; 24.03	2002/2003 (1 year)	wooden panels
**6 Oban- Scotland**	56.41; −5.80	2002/2003 (1 year)	wooden panels
**7 Roskilde- Denmark**	55.63; 12.08	2002/2003 (1 year)	wooden panels
**8 Mouth of River Prerowstorm- Germany**	54.41; 12.61	2002 (1 year)	wooden panels at shipwreck site
**9 Kiel- Germany**	54.36; 10.15	2002/2003 (1 year)	wooden panels
**10 Haren- Netherlands**	53.48; 6.76	2002/2003 (1 year)	wooden panels
**11 Near Texel- Netherlands**	53.06; 4.90	2002 (1 year)	wooden panels at shipwreck site
**12 Yerseke- Netherlands**	51.53; 3.98	2002/2003 (1 year)	collecting panels
**13 Rye- England**	50.93; 0.76	2001 (1 day)	old wooden piles
**14 Portsmouth- England**	50.79; −1.02	2002/2003; 2003/2004 (2 years)	wooden panels
**15 Lyme Regis- England**	50.72; −2.93	2004 (1 day)	samples of local wooden structures
**16 Bournemouth- England**	50.71; −1.87	2003 (1 day)	old wooden piles
**17 Swanage- England**	50.60; −1.95	2001 (1 day)	samples from the old pier
**18 Toulindac- France**	47.60; −2.87	2008 (1 day)	wooden structures
**19 Golfe du Morbihan- France**	47.56; −2.79	2009 (1 day)	wooden structures
**20 Berder- France**	47.55; −2.48	2008 (1 day)	wooden structures
**21 Penerf- France**	47.50; −2.61	2009 (1 day)	wooden structures
**22 Rovinj- Croatia**	45.08; 13.63	2002/2003 (1 year)	wooden panels
**23 Banyuls-sur-mer- France**	42.48; 3.12	2009 (1 day)	wooden structures
**24 Bartin- Turkey**	41.68; 32.22	2002/2003 (1 year)	wooden panels
**25 Viana do Castelo- Portugal**	41.74; −8.88	2009	wooden structure
**26 Aveiro- Portugal**	40.62; −8.70	2004	wooden structure
**27 Praia da Vitória, Terceira- Azores**	38.71; −27.04	2002/2003 (1 year)	wooden panels
**28 Porto Martins, Terceira- Azores**	38.68; −27.05	2001 (1 day)	wooden structures
**29 Lisbon- Portugal**	38.67; −9.20	1990/2006 (16 years)	old wooden piles
**30 Angra do Heroísmo, Terceira- Azores**	38.65; −27.21	2001 (1 day)	wooden structures
**31 Mosteiros, São Miguel- Azores**	37.88; −25.80	2011 (1 week)	wooden structure?
**32 Olhão- Portugal**	37.00; −7.79	2002/2003; 2003/2004 (2 years)	wooden panels
**33 Mersin- Turkey**	36.80; 34.64	2002/2003; 2006/2007 (2 years)	wooden panels
**34 Ka- Turkey**	36.19; 29.63	2010 (1 week)	samples from the shipwreck

Site name; geographic coordinates (decimal degrees); time and duration of surveys; and type of structure from which limnoriids where collected from.

### Ecological requirements of wood boring limnoriids

**Figure 4:**
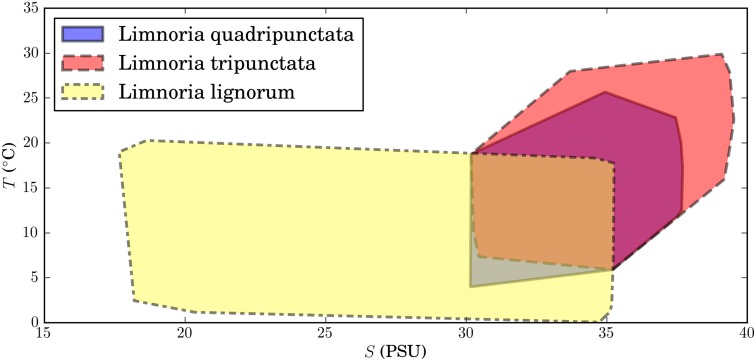
Distribution of the three limnoriid species in salinity-temperature (*S–T*) space. The minimum convex polygon encompassing all data points represent the climatic niche of each species.

The two wood boring limnoriid species that tolerate the lowest temperatures are *Limnoria lignorum* and *L. quadripunctatata,* with limits of 1° and 4°C, respectively (Fig. 4). Their highest temperature limits are 20 and 25°C, respectively. *Limnoria lignorum* tolerates lower salinities (∼17 PSU) than *L. quadripunctata* (∼30 PSU), but the upper salinity limits registered in this study are similar for both species, 36 and 37 PSU, respectively. Of the three species, *L. tripunctata* showed the highest requirements of temperature (11–30°C) and salinity (31–39 PSU) (Fig. 4).

The temperature and salinity requirements of *Limnoria borealis*, *L. carinata* and *L. tuberculata* were not characterized because of the low number of occurrences of these three species in European coastal waters.

## Discussion

### Diversity and origin of wood boring limnoriids in European coastal waters

From our field surveys, we collected only *Limnoria lignorum*, *L. quadripunctata* and *L. tripunctata*. These species were found either in wooden collecting panels or in fixed wooden maritime structures in the areas surveyed (Table S1). Therefore, they are considered part of the established fauna in European coastal waters, according to the criteria defined by Turner [31] for marine wood borers and the general definition of established species in the CIESM atlas of exotic species in the Mediterranean, also used by [32] (please see Materials and methods for the definition of established species). *Limnoria borealis* was found in the continental shelf of Iceland at depths between 170–260 m, and apparently it did not get established in wooden structures in Icelandic waters [30]. As far as the authors are aware, it has never been reported established in Europe.

The taxonomic status of *Limnoria carinata* and *L. tuberculata* was considered uncertain [33], because of the morphological similarities between these species, and *L. quadripunctata* and *L. tripunctata*, respectively. This led Esakova [34] to synonymize *L. tuberculata* and *L. tripunctata* and consider the former a senior synonym of the latter, which was considered erroneous by Menzies [35]. To clarify the subject, interbreeding experiments were conducted between specimens of the two putative species from populations of different geographical locations. These experiments showed that *L. tripunctata* and *L. tuberculata* are valid species [35]. Later, Cookson [36] found enough morphological differences between the two species and re-described *L. tuberculata,* to make it easier to distinguish it from *L. tripunctata*.

In spite of the taxonomic work done on *Limnoria tuberculata*, it is not clear whether or not this species is established in European waters because its last known occurrence in Europe (Aegean Sea) dates from 1995 [29]. Morphologically, *L. carinata* and *L. quadripunctata* are very similar, which casted doubts on the taxonomic status of the former species [33], [36]. Kühne [33] suggested that these species should be synonymized. However, recent work by Castelló [2] on the taxonomy and systematics of limnoriids from European waters showed that *L. carinata* is a valid species. *Limnoria carinata* recruited to pine collectors in the Harbour of Naples, and it was also found in driftwood in La Spezia, Italy [27]. As far as the authors are aware, it has never been reported anywhere else in European waters. Therefore, its status is considered uncertain because there is only one record of the species (Naples), where it can be considered established.

The origin of wood boring limnoriids in Europe is still unknown. Menzies [3] and Jones [37] considered *L. lignorum* to be native of Europe (Netherlands and Britain, respectively). Later authors, however, considered *L. lignorum* cryptogenic (e.g [38]) because during the long voyages of discovery, wooden vessels may have significantly changed the distribution of marine organisms, particularly those with direct development, such as limnoriids [24]. However (Carlton, pers. com.) considers that *L. lignorum* is probably native to both the Pacific and the Atlantic, northern hemisphere, whereas *L. quadripunctata* and *L. tripunctata* are most probably introduced in Europe, and the latter may have originated in the southern hemisphere [39], [40]. The introduction of *L. quadripunctata* and *L. tripunctata* in Europe might, however, have preceded their description. For instance, examination of preserved material from Plymouth waters, southern England, showed that *L. quadripunctata* had been present in the area since at least 1930 [37]. Nevertheless, until there is definitive evidence on their origin, for instance molecular data, *L. lignorum*, *L. quadripunctata* and *L. tripunctata* should be considered cryptogenic, as is the case for most teredinid species established in European waters [25].

### Factors affecting the presence/absence of wood boring limnoriids

At several sites surveyed in this study, limnoriids were not present. One of the reasons might be that the environmental conditions were not adequate. Another might be that wood was not as available, as many coastal areas, such as the dykes of The Netherlands have been ‘petrified’ [41] and in southern Europe concrete and steel have been largely used to build sea defences [42]. In addition, the particular habitat limnoriids inhabit, wood, makes these organisms inconspicuous in general surveys, and their presence might have gone unnoticed. Usually, the presence of wood boring limnoriids is noticed only in situations where wooden structures show the typical superficial attack caused by these organisms [7].

Colonisation of new wood occurs during the migratory phase, but this stage is unlikely to be responsible for long-distance dispersal (LDD) of limnoriid species. Limnoriids have limited swimming capability and, unlike teredinids, do not possess the larval stage, which plays a very important role in dispersal of teredinids [43], [44]. Therefore, it is unlikely that these organisms can achieve any significant dispersal by swimming [44]. This limitation undoubtedly affects their dispersal to distant areas with suitable environmental conditions [7]. In the past, the main vectors for short-distance dispersal (SDD) and long-distance dispersal (LDD) of limnoriids were probably the hulls of wooden ships that crossed the oceans [7], [45], [46]. Nowadays this type of vector plays no significant part in the distribution of these organisms, as few wooden vessels exist in present days. Therefore other mechanisms may play a role on the dispersal of these species. In SDD, for instance along shelf corridors, rafting (in driftwood) may provide a particularly important dispersal means to wood-boring limnoriids as it does for many other organisms, including algal-boring limnoriids [47], [48]. LDD and colonisation of new areas may also be achieved by means of rafting in driftwood transported by currents if, as is hypothesised for algal-boring limnoriids [49], wood-boring limnoriids are able to reproduce successfully during rafting. However, although limnoriids were observed rafting considerable distances (400 to 600 km) in kelp holdfasts [50], there is no evidence, so far, of limnoriids surviving LDD, rafting across ocean basins. In fact, estuarine and harbour wood-boring species have never been reported rafting in ocean currents [39].

Other important vectors of transport of alien species include shipping activities (recreational and commercial). Ships can transport such species in ballast water, ballast sediment and fouling of numerous external surfaces, and of sea-chests (protected areas built in the hull below the waterline, where the pipes for sea-water intake, engine cooling and fire-fighting are located) [51], [52], [53], [54], [55]. The transport of wood boring limnoriids either in ballast water or ballast water sediment cannot be discounted, but the sea-sieves or strainers located between the sea-chests and the pumps are designed to retain objects larger than 5 mm [55] and therefore it is unlikely that pieces of wood containing limnoriids could enter the ballast water tanks. It would be possible for the organisms to enter this area during nocturnal migrations [56]. However, so far, limnoriids have never been found in ballast water or in ballast water sediment (Briski, pers. com., Carlton, pers. com., Gollasch, pers. com.). Evidence has been emerging, however, that sea-chests may explain the transport of many organisms that cannot enter the ballast water tanks [57], [58]. Their size varies with vessels as well as the diameter of the holes in the steel grille that protects them (dimensions of the order of 15–35 mm wide). Thus, small pieces of wood may be able to enter these areas that will become vectors for organisms such as wood-boring limnoriids. Although limnoriids have never been reported in sea-chests, this might be due to the fact that these areas are usually inaccessible to surveys, except during slipping and dry-docking, making research more difficult [55]. This type of vector might be responsible for the transport of limnoriids to remote areas such as the Azores. Once in an area with available wood, and favourable environmental conditions, these organisms can infest the new wood immediately and compete effectively for instance with teredinids [1]. The competitive capacity of limnoriids is probably related to extended parental care after hatching, when limnoriids are thought not to have yet developed full boring capacity [24]. The parental care maximises juvenile survival rates and therefore rapid infestation of the wood in which they borrow.

### Biogeography of limnoriid species in European coastal waters

*Limnoria lignorum* is the wood boring limnoriid species with the most northern distribution in European coastal waters. This is probably related to the range of water temperatures tolerated by this species. In the present study we inferred that *L. lignorum* tolerates temperatures ranging from 1°C and 20°C. The minimum temperature inferred in this study is much lower than the lower limit of temperature previously reported for this species (10°C) by Kühne [59]. In laboratory conditions, it was observed that below 5°C the survival of the species is precarious and the organisms in these conditions are nearly dormant [60]. However, experiments carried out in Norway showed that *L. lignorum* can withstand temperatures as low as −4°C without suffering ill effects. In addition, animals thawed out from a state of suspended animation were able to swim out of their burrows, suggesting that they are capable of migrating [61]. Thus, in natural conditions the dormant state is probably an adaptation to survive low winter temperatures in areas such as Norway and Iceland.

The reported wider distribution range of *L. lignorum* in Europe relatively to *L. quadripunctata*, and *L. tripunctata* may be explained partially by the wider range of salinities tolerated by *L. lignorum* (17–35 PSU). Indeed, our results indicate that *L. quadripunctata* and *L. tripunctata* are stenohaline (Fig. 4). Another possible explanation might be that until the mid-twentieth century *L. lignorum* was the only wood-boring limnoriid species described and, therefore, some identifications previous and soon after the description of *L. quadripunctata* and *L. tripunctata* were probably erroneous (e.g. [12]).

*L. lignorum* used to be common in southern England [17], [37], [62] (see Fig. 3 and TableS1). However, in the present study the southern distribution limit of *Limnoria lignorum* was Yerseke, southern Netherlands. In the past this species was also reported in Cherbourg, France, where it caused extensive damage in the 19^th^ century (Hubrecht et al., 1886 in [9]), Coruña-Galicia, Spain [63], Cadiz [64] and in the Alboran Island and Tarifa [65]. The last three publications, however, do not provide evidence of *L. lignorum* being part of the local established fauna in those areas. Nevertheless, all these records seem to indicate a poleward shift of the species. This observations agree with long-term monitoring and global meta-analysis, which shows significant poleward range shifts of species [66], [67], averaging 50 km per decade [67]. These poleward shifts are also associated with increase in abundance of southern species of plankton [66] and fishes [69], [70].

Climate seems to have affected range shifts of species and also competition between them [71]. In our field surveys, the limnoriid species that occurred in 4 out of the 5 sites surveyed in southern England was *L. quadripunctata* (Fig. 3). In the fifth site limnoriids were not present in the wooden structures surveyed. *L. lignorum* may have been outcompeted by *L. quadripunctata* in these areas, where warm water temperatures might have a negative effect in the cold water *L. lignorum*, similarly to other species in southern England. *Semibalanus balanoides* also suffered climate-mediated competitive exclusion [72]. The warm water has a significant adverse effect on *S. balanadoides* (a cold-water species), but not on *chthamalus* species (warm water species). The latter compete with the former and are now the dominant species in southwest England [71].

Since its description in 1949, *L. quadripunctata* was reported occurring only in the coastal waters of The Netherlands [11], UK (e.g. [17], [37]), and later in the Atlantic coast of France [73]. However, the distribution of *L. quadriunctata* obtained from our field surveys indicates an expansion of its range further south. The species was found established in the Atlantic and Mediterranean coasts of France ([74], this study), in the Tagus Estuary [42], in Viana do Castelo, northern Portugal (this study), and in Mosteiros, São Miguel, Azores (this study). In the Tagus estuary and in São Miguel, Azores, *L. quadripunctata* was found occurring in sympatry with *L. tripunctata* (Fig. 4), and therefore competition probably occurs between these two species.

In European waters, *L. tripunctata* was reported before 2000 mainly from the UK, where several surveys on marine wood borers were undertaken [17], [37], [75], and from France [73]. It was also reported from Lisbon, Portugal [12], [42] but occurring there only sporadically, and in low numbers [42]. However, in the present study *L. tripunctata* was not found in any of the 5 test sites surveyed in southern England or in the Atlantic coast of France (see table 1 for sites surveyed). Thus, a range contraction seems to have occurred along its northern limit. This might be due to climate-mediated competition with *Limnoria quadripunctata*, which is common in southern England and along the Atlantic coast of France. In the present study no limnoriids were found in the collecting panels exposed in Rovinj, Croatia, although the species was reported occurring in the Adriatic before 2000 (see Table S1 and Fig. 3), which may mean also a range contraction in the Mediterranean. Similarly, records from the present study show that *L. tripunctata* is more widespread in Portuguese waters and also in eastern Mediterranean than previous reports seemed to indicate. Indeed, this species was found to occur in Porto [76], Aveiro, Olhão, Praia da Vitória, Terceira and Mosteiros, São Miguel, Azores and also in Mersin, southern Turkey (Table S1). In Olhão and Terceira, *L. tripunctata* was the most abundant and destructive wood boring species [21]. In both areas, temperature and salinity of the water might be more favourable to the survival of this species, triggering an increase in the numbers of specimens occurring in these areas, similarly to what has been observed in other species in southern waters [68], [69], [70]. This probably leads to a higher competition ability of *L. tripunctata*.

## Conclusions

Of all three wood boring limnoriid species established in European waters, a range extension appears to have occurred in *Limnoria quadripunctata* and *L. tripunctata*, whereas occurrence records of *L. lignorum* (Table S1) seem to indicate a poleward contraction of the species distribution. Indeed *L. quadripunctata* seems to be very abundant in areas such as southern England and France, and it may be out-competing other limnoriid species in these areas. *Limnoria tripunctata* on the other hand seems to be particularly well adapted to Portuguese waters (e.g. Olhão and Terceira, Azores), where its destructive activity is rivalling that of teredinids [21], [42], [76]. In other areas, such as Venice, limnoriids also seem to cause considerable destruction to the wooden structures in the lagoon (Borges, pers. obs.). The destructive activity of limnoriids in European coastal waters makes it important to continue monitoring their activity in European waters.

The works of Cookson [36] and Castelló [2] were of great importance to infer the taxonomic status (accepted) of *Limnoria tuberculata* and *L. carinata*, respectively. However, these species are still difficult to identify based only on morphology, due to the fact that morphological characters can be easily damaged. This may lead to misidentifications, with consequences in the study of the biogeography of these species. Further surveys should be carried out, particularly in the Mediterranean and the use of molecular markers should be considered, in addition to morphological identification. This will improve taxonomic resolution and therefore elucidate the biogeography of these species in Europe, as has been done for European teredinid species [46], [77].

## Materials and Methods

We sampled a total of 34 sites in European coastal waters between 2001 and 2011 (Fig. 1; Table 1). From May 2002 to May 2003 we carried out a survey in 15 sites, selected to represent different seawater temperature and salinity conditions in Europe. At each site, six replicate panels of *Pinus sylvestris* L. (20×10×2 cm), were used as baits to collect wood-borers (for full methodology please refer to [25]). In addition, limnoriids were collected opportunistically in a number of maritime wooden structures (see Table 1). This study did not involve vertebrates, endangered or protected species. No specific permissions were required for deploying the test panels in the areas surveyed as they are not protected areas. Specimens collected during the field surveys were identified morphologically using the descriptions of Castelló [2], Menzies [3], Holthuis [11], Kühne [33], and Cookson [36]. In addition to the data obtained from field surveys, we gathered records of occurrence data of established limnoriid species from a comprehensive review of literature dating back to the 1900s including: specific works that refer to the distribution of limnoriids [2], [3], [6], [10], [11], [12], [13], [14], [17], , faunal compilations [28], [29], [32], [63], [64], [65], unpublished reports [75], and online databases (e.g. [78], [79], [80]). We compiled a database containing the occurrence data, including the locality name, geographic coordinates and year of occurrence (Table S1). Care was taken not to include dubious records (e.g. a record of *Limnoria lignorum* in Portugal in [81]) or records of species found only in driftwood. All occurrences before 2000 were mapped as past distributions, while occurrences since 2000 were mapped as recent distribution.

We considered as established, wood-boring species with a minimum of two published records either from different localities or from different time periods [82]. In addition, there should be evidence that specimens are able to breed successfully and grow to maturity in a given area. This can be determined either using wooden collecting panels or by collecting specimens from local fixed wooden structures [31].

To determine the climatic conditions suitable for the survival of limnoriid species, the distribution of each established species was represented in salinity-temperature (*S-T*) space. A climatic envelope was then defined as the area enclosed by the minimum convex polygon encompassing all data points. Sea surface temperature (SST) and sea surface salinity (SSS) were obtained from a global hybrid dataset compiled by [25]. This hybrid dataset was based primarily on the global environmental dataset in BIO-ORACLE [83], using the long-term variation for salinity provided by the Research Archive (RDA) [84]. The resolution of coastal areas in the North and Baltic Seas in the hybrid dataset was further improved by including the numerical model output from the Coastal Observation System for North and Arctic Seas (COSYNA) [85]. For further details on the methodology used, please refer to [25].

## Supporting Information

Table S1 **Occurrence of wood boring limnoriids (Limnoriidae) in European coastal waters.**
(PDF)
