# Supplementary material for: Biogeography of Wood-Boring Crustaceans (Isopoda: Limnoriidae) Established in European Coastal Waters
Source: PLoS One. 2014 Oct 14;9(10):e109593. doi: 10.1371/journal.pone.0109593 (PMC4196924; doi:10.1371/journal.pone.0109593)
Supplement: Table S1 — Occurrence of wood boring limnoriids (Limnoriidae) in European coastal waters. (PDF) [file pone.0109593.s001.pdf]

Table 2- Occurrence of wood-boring limnoriids (Limnoriidae) in European coastal waters.

Species/ authority	Location	Coordinates decimal degrees	Source
Limnoria lignorum (Rathke, 1799)	Hammerfest Norway	70.66; 23.67	Somme, 1940
	Torsvåg-Norway	70.26; 19.54	Somme, 1940
	Tromso Sound-Norway	69.66; 19.01	Somme, 1940
	Reykjavik-Iceland	64.15; -21.89	Saemundsson, 1937; Stepensen, 1937; Svavarsson, 1982; May 2002 to May 2003 (this study)
	Vestmanneyjar-Iceland	63.44; -20.26	Saemundsson, 1937
	Keflavík-Iceland	64.00; -21.54	Saemundsson, 1937
	Hafnarfjörour-Iceland	64.06; -21.96	Saemundsson, 1937
	Stykkishólmur-Iceland	65.07; -22.72	Saemundsson, 1937
	Vatneyri-Iceland	65.60; -23.59	Saemundsson, 1937
	Bíldudalur-Iceland	65.68;- 23.59	Saemundsson, 1937
	Ísafjörour-Iceland	66.06; -23.12	Saemundsson, 1937
	Stekkeyri-Iceland	66.33; -22.87	Saemundsson, 1937
	Hrísey-Iceland	65.98; -18.39	Saemundsson, 1937
	Raufarhöfn-Iceland	66.45; -15.94	Saemundsson, 1937
	Vopnafjörour-Iceland	65.75; -14.81	Saemundsson, 1937
	Norofjörour-Iceland	65.13; -13.73	Saemundsson, 1937
	Djúpavogur-Iceland	64.11; -21.94	Saemundsson, 1937
	Trondheim-Norway	63.41; 10.41	Santhakumaran and Sneli, 1978; Santhakumaran, 1984; Santhakumaran and Sneli, 1984; May 2002 to May 2003 (this study)
	Floro- Norway	61.60; 5.03	Jones et al., 1972
	Lyness-Scotland	58.83; -3.19	Hall and Saunders, 1967
	Flødevigen-Norway	58.42; 8.75	Somme, 1940
	Kristineberg Biological Station-Sweden	58.03; 11.05	Henningsson and Norman, 1980; Westin, pers. com.; May 2002 to May 2003 (this study)
	Aultbea-Scotland	57.84; -5.58	Hall and Saunders, 1967
	Loch Linnhe-Scotland	56.81; -5.11	Jones, 1963

Table 2 (continued)- Occurrence of wood-boring limnoriids (Limnoriidae) in European coastal waters.

Limnoria lignorum (Rathke, 1799)	Harbour of Hirshals-Denmark	57.59; 9.97	Kramp, 1937
	Firth of Forth (Methil)-Scotland	56.18; -3.01	Ritchie, 1927
	Gareloachhead-Scotland	56.07; -4.83	Hall and Saunders, 1967
	Millport-Scotland	56.00; -3.05	Jones, 1963; Hall and Saunders, 1967
	Blyth-England	55.12; -1.49	Jones, 1963; Hall and Saunders, 1967
	West Hartlepool-England	54.68; -1.19	Hall and Saunders, 1967
	Belfast-Northern Ireland	54.64; -5.89	Hall and Saunders, 1967
	Ramsey-Isle of Man	54.31; -4.37	Jones, 1963
	Helgoland- Germany	54.17; 7.89	Jones et al., 1972
	Barrow-England	54.09; -3.22	Jones, 1963
	Fletwood Harbour-England	53.92; -3.03	Rattray, 1920; Jones, 1963
	River Humber (Lincolnshire)-England	53.58; 0.08	Cartwright, 1920
	Ballynakill Harbour (Galway)-Ireland	53.57; -9.26	Tattersall, 1906
	Port Twyn Mawr, Anglesey-Wales	53.27; -4.61	Jones, 1963
	Tal-Y-Foel, Anglesey-Wales	53.14; -430	Jones, 1963
	Grimsby-England	53.57; -0.05	Jones, 1963; Hall and Saunders, 1967
	Corton nr Lowerstoft Egland	52.50; 1.75	Jones, 1963
	Lowerstoft Harbour-England	52.47; 1.75	Jones, 1963
	Milford Docks-Wales	51.71; -5.04	Jones, 1963
	Pembroke-England	51.69; -4.94	Jones, 1963

Table 2 (continued)- Occurrence of wood-boring limnoriids (Limnoriidae) in European coastal waters.

Limnoria lignorum (Rathke, 1799)	Burnham on Crouch-England	51.62; 0.80	Jones, 1963
	Port Talbot-Wales	51.58; -3.81	Hall and Saunders, 1967
	Yerseke-Netherlands	51.53;3.98	May 2002 to May 2003 (this study)
	Portishead (Bristol Channel)-England	51.49; -2.72	Purchon, 1938
	Cardiff-Wales	51.45; -3.16	Hall and Saunders, 1967
	Barry-Wales	51.39; -3.25	Jones, 1963; Hall and Saunders, 1967
	Kosksijde- Belgium	51.22; 2.62	Holthuis, 1950
	Dover-England	51.11; 1.31	Wilson, 1920; 1967; Hall and Saunders, 1967
	Southampton-England	50.89; -1.40	Eltringham & Hockley, 1958; Jones, 1963; Hall and Saunders, 1967
	Plymouth-England	50.86; -4.13	Jones, 1963; Hall and Saunders, 1967
	Shoreham-England	50.82; -0.25	Jones, 1963; Hall and Saunders, 1967
	Portsmouth-England	50.79; -1.02	Hall and Saunders, 1967; Jones et al., 1972
	Hayling Harbour	50.78; -0.93	Eltringham, 1957
	Wimereux- France	50.77; 1.60	Müller, 2004
	Cowes, Isle of Wight-England	50.76; -1.29	Jones, 1963
	Lymington-England	50.73; -1.51	Jones, 1963
	Poole-England	50.69; -1.92	Hall and Saunders, 1967
	Brixham-England	50.39; -3.51	Hall and Saunders, 1967
	Newton Ferrers-England	50.30; -4.07	Hall and Saunders, 1967
Limnoria quadripunctata Holthuis, 1949	Ramsey-Isle of Man	54.31; -4.37	Jones, 1963
	Barrow-England	54.09; -3.22	Jones, 1963
	Dublin-Southern Ireland	53.33; -6.18	Hall and Saunders, 1967
	Dun Laoghaire-Southern Ireland	53.29; -6.12	Jones, 1963
	Noordwijk-Netherlands	52.23; 4.03	Holthuis, 1949
	Cork- Ireland	51.79;-8.26	Grave and Holmes, 1988

Table 2 (continued)- Occurrence of wood-boring limnoriids (Limnoriidae) in European coastal waters.

Limnoria quadripunctata Holthuis, 1949	Milford Docks-Wales	51.71; -5.04	Jones, 1963
	Swansea-Wales	51.61; -3.94	Jones, 1963; Hall and Saunders, 1967
	Port Talbot-Wales	51.58; -3.81	Jones, 1963
	Lough Hyne- Ireland	51.50; -9.30	Grave & Holmes, 1998
	Cardiff-Wales	51.45; -3.16	Hall and Saunders, 1967
	Ilfracombe Harbour-England	51.21; -4.11	Jones, 1963
	Dover-England	51.11; 1.31	Jones, 1963
	Southampton-England	50.89; -1.40	Eltringham and Hockley, 1958; Jones, 1963; Hall and Saunders, 1967
	Plymouth-England	50.86; -4.13	Hall and Saunders, 1967
	Shoreham-England	50.82; -0.25	Jones, 1963; Hall and Saunders, 1967
	Callshot, Southampton-England	50.81; -1.31	Jones 1963
	Yarmouth (Isle of Wight)-England	50.72; -1.54	Cragg, pers. com.
	Portsmouth-England	50.79; -1.02	May 2002 to May 2003; 2004, 2005 (this study)
	Hayling Harbour	50.78; -0.93	Eltringham, 1957
	Cowes, Isle of Wight-England	50.76; -1.29	Jones, 1963
	Lymington- England	50.73; -1.51	Jones, 1963
	Lyme Regis-England	50.72; -2.93	2004 (study)
	Bournemouth-England	50.71; -1.87	2003 (study)
	Poole-England	50.69; -1.92	Hall and Saunders, 1967
	Weymouth- England	50.61; -2.44	Eltringham, 1957
	Swanage-England	50.60; -1.95	2001 (study)
	Padstow- England	50.54; -4.93	Jones, 1963
	Brixham-England	50.39; -3.51	Jones, 1963; Hall and Saunders, 1967
	Plymouth-England	50.86; -4.13	Jones, 1963
	Newton Ferrers-England	50.30; -4.07	Hall and Saunders, 1967

Table 2 (continued)- Occurrence of wood-boring limnoriids (Limnoriidae) in European coastal waters.

Limnoria quadripunctata Holthuis, 1949	Falmouth Docks-England	50.15; -5.04	Jones, 1963
	St Helier-Jersey	49.17; -2.11	Jones, 1963
	Golfe du Morbihan-France	47.00; -2.00	2009 (this study)
	Baie de Villaine- France	47.00; -2.00	2009 (this study)
	La Rochelle-France	46.15; -1.15	Jones et al., 1972
	Trieste-Italy	45.64; 13.75	Menzies & Becker, 1957
	Banyuls-sur-mer	42.28; 3.07	2008 (this study)
	Viana do Castelo-Portugal	41.68; -8.84	2009 (This study)
	Port of Leixôes-Portugal	41.18; -8.70	Lopes et al., 2013
	Tagus Estuary-Portugal	38.67; -9.20	2007 (this study)
	São Miguel- Azores	37.92; -25.80	2011 (this study)
Limnoria tripunctata Menzies, 1951	Swansea-Wales	51.61; -3.94	Jones, 1963; Hall and Saunders, 1967
	Cardiff-Wales	51.45; -3.16	Hall and Saunders, 1967
	Barry-Wales	51.39; -3.25	Jones, 1963; Hall and Saunders, 1967
	Southampton-England	50.89; -1.40	Eltringham and Hockley, 1958; Jones, 1963; Hall and Saunders, 1967
	Shoreham-England	50.82; -0.25	Hall and Saunders, 1967
	Callshot, Southampton-England	50.81; -1.31	Jones 1963
	Portsmouth-England	50.79; -1.02	Hall and Saunders, 1967
	Cowes, Isle of Wight-England	50.76; -1.29	Jones, 1963
	Lymington- England	50.73; -1.51	Jones, 1963
	Poole-England	50.69; -1.92	Hall and Saunders, 1967
	La Rochelle-France	46.15; -1.15	Jones et al., 1972
	Trieste-Italy	45.64; 13.75	Menzies & Becker, 1957
	Venice lagoon-Italy	45.44; 12.30	Menzies & Becker, 1957

Table 2 (continued)- Occurrence of wood-boring limnoriids (Limnoriidae) in European coastal waters.

Limnoria tripunctata Menzies, 1951	Harbour of Naples-Italy	40.83; 14.25	Menzies & Becker, 1957
	Port of Leixões-Portugal	41.18; -8.70	Lopes et al., 2013
	Aveiro- Portugal	40.62; -8.70	2004 (this study)
	Terceira- Azores	38.71; -27.04	May 2002 to May 2003 (this study)
	Tagus Estuary-Portugal	38.67; -9.20	Franco, 1962; Jones et al., 1972; 2007 (this study)
	Lipari-Italy	38.46; 14.96	Jones et al., 1972
	São Miguel- Azores	37.92; -25.80	2011 (this study)
	Olhao Harbour-Portugal	37.00; -7.79	May 2002 to May 2003 (this study)
	Mersin Bay-Turkey	36.80; 34.64	May 2002 to May 2003 (this study); Sivrikaya et al., 2009
	Iskenderun-Turkey	36.59; 36.17	Sen et al. , 2010
	Finike-Turkey	36.29; 30.14	Sen et al. , 2010

References

Eltringham SK (1957) The biology of the wood-boring isopod *Limnoria* from Southampton waters. PhD thesis. University of Southampton, Southampton, United Kingdom.

Eltringham SK, Hockley AR (1958) Coexistence of three species of the wood-boring Isopod *Limnoria* in Southampton water. Nature 4624: 1659–1660.

Franco SE (1962) Os ataques de xilófagos marinhos em estruturas de madeira submersas. Ensaios de durabilidade natural de ensaios de madeira-II. *Separata da Revista Agronómica*, XLV:Tomo III.

Grave S, Holmes JMC (1998) The distribution of marine Isopoda (Crustacea) in Lough Hyne. Biology and Environment, Proceedings of the Royal Irish Academy 98(B): 23-30.

Hall GS, Saunders RG (1967) Incidence of marine borers round Britain's coasts. Timber Research and Development Association (TRADA).

Henningsson B, Norman E (1980) A marine borer test with water-borne preservatives. Proceedings of the International Research Group on Wood Preservation IRG/WP/80-452. 5 p.

Holthuis LB (1949) The Isopoda and Tanaidacea of the Netherlands, including the description of a new species of *Limnoria*. Zoologische Mededelingen 30: 163-190.

Holthuis LB (1950) Isopodes et Tanaidacés marins de la Belgique; remarques sur quelques espèces de la zone méridionale de la Mer du Nord. Med K Belg Inst Nat Wet 26(53): 1-19

Jones LT (1963) The geographical and vertical distribution of British *Limnoria* [Crustacea: Isopoda]. J Mar Biol. Ass UK 43, 589-603.

Jones EBG, Turner RD, Furtado SEJ, Kühne H (1972) Results of an international cooperative research programme on the biodeterioration of timber submerged in the sea. Mat Org **7**: 93–118.

Le Roux A (2009) Aperçu de la faune des xylophages marins du golfe du Morbihan. Bull Soc Sci Nat Ouest Fr 31(2), 75–83.

Lopes DB (2013) Technological improvement of Portuguese pinewood by chemical modification. PhD thesis. Georg-August-University Göttingen, Germany.

Menzies R J, Becker G (1957) Holzzerstörende *Limnoria*-Arten (Crustacea, Isopoda) aus dem Mittlmeer mit Neubeschreibung von *L. carinata*. Z. Angew. Zool 44: 85–92.

Purchon R (1938) Studies on the biology of the Bristol Channel II. An ecological study of the beach and the dock at Portishead. Proceedings of the Bristol Naturalists´Society 8(3): 311–329.

Rattray D (1920) Fletwood. Report of the Comm. [deter. structures]. Institution of Civil Engineering (London). pp 97–101.

Ritchie J (1927) An account of the destruction of Metbil dock gates by marine organisms. Scottish Naturalist 164: 37–44.

Saemundsson B (1937) Icelandic Malacostraca in the museum of Reykjavik. Societas Scientiarum Islandic Vol. 20.

Santhakumaran LN, Sneli J-A (1978) Natural resistance of different species of timber to marine borer attack in the Trondheimsfjord (Western Norway). Interantional Research Group on Wood Preservation IRG/WP/435.

Santhakumaran LN, Sneli J-A (1984) Studies on the marine fouling and wood-boring organisms of the Trondheimsfjord (western Norway). *Gunneria* 47, 7–30.

Sen S, Sivrikaya H, Yalcin M, Bakir AK, et al. (2010) Fouling and boring organisms that deteriorate various European and tropical woods at Turkish seas. African Journal of Biotechnology 9(17): 2566–2573

Sømme O (1940) A study of the life history of the gribble *Limnoira lignorum* (Rathke) in Norway. NYTT Magasin for Nautuvuidenskapene 81: 145–205.

Stephensen K (1937) Marine Isopoda and Tanaidacea. The Zoology of Iceland III, Part 27.

Svavarsson J (1982) *Limnoria borealis* (Isopoda, Flabellifera) and its commensal, Caecijaera borealis (Isopoda, Aselota), found in Icelandic waters. Sarsia 67: 223–226.

Tattersall D (1906) The marine fauna of the coast of Ireland. V. Isopoda. Report of the sea amd inland fisheries of Ireland for 1904, Dublin. pp 53–142.

Wilson M (1920a) Dover. Rep Comm. [Deter. structures]. Institution of Civil Engineer (London). pp 93–94.

Wilson M (1920b) Southampton. Rep Comm. [Deter. Structures]. Institution of Civil Engineer (London). pp 150–151.
